# Subclinical attention-deficit hyperactivity disorder symptoms and unhealthy lifestyle behaviours

**DOI:** 10.1192/bjo.2024.785

**Published:** 2024-10-03

**Authors:** Sara Gostoli, Giulia Raimondi, Paola Gremigni, Chiara Rafanelli

**Affiliations:** Department of Psychology ‘Renzo Canestrari’, University of Bologna, Italy

**Keywords:** Attention-deficit hyperactivity disorder, adolescence, psychological well-being, subclinical symptoms, unhealthy lifestyle behaviours

## Abstract

**Background:**

Literature emphasises the importance of identifying and intervening in the adoption of unhealthy lifestyle behaviours (ULBs) during adolescence at an early stage, to mitigate their long-term detrimental effects. Among the possible associated factors contributing to ULBs, attention-deficit hyperactivity disorder (ADHD) has been shown to play an important role. However, little is known about ADHD subclinical manifestations.

**Aims:**

The present study aimed to bridge the gap in the literature and shed light on the relationship between subclinical ADHD and early adoption of ULBs during adolescence. Through a clinimetric approach, prevalence of ULBs, severity of ADHD symptoms and psychosocial factors (i.e. allostatic overload, abnormal illness behaviour, quality of life, psychological well-being) were investigated among adolescents. The associations between different degrees of ADHD, ULBs and psychosocial factors were also explored.

**Method:**

This multicentre cross-sectional study involved 440 adolescents (54.5% females; mean age 14.21 years) from six upper secondary schools. Participants completed self-report questionnaires on sociodemographic characteristics, ULBs, ADHD symptoms and psychosocial factors.

**Results:**

The most common ULBs were energy drinks/alcohol consumption and problematic smartphone use. Of the sample, 22% showed subclinical ADHD and 20.2% showed clinical ADHD. The subclinical ADHD group showed several ULBs (i.e. altered mindful eating, impaired quality of sleep, problematic technology use) and psychosocial factors, akin to those of ADHD group and different from peers without ADHD symptoms.

**Conclusions:**

Since subclinical ADHD manifestation is associated with ULBs, similarly to clinical ADHD, identifying subthreshold symptoms during adolescence is crucial, as it could improve health-related outcomes in adulthood across different domains.

Adolescence is characterised by rapid biopsychosocial changes and the adoption of unhealthy lifestyle behaviours (ULBs) influenced by social pressure from peers.^1^ Social restrictions during the COVID-19 pandemic have exacerbated adolescents’ poor dietary habits, physical inactivity^2^ and technology addiction.^3^ Moreover, adopting ULBs during adolescence significantly predicts the development of psychiatric disorders^4^ and non-communicable diseases (e.g. cardiovascular diseases, cancer, diabetes) in adulthood,^5^ which are mainly caused by modifiable environmental factors and account for 74% of deaths worldwide.^6^

Therefore, it is crucial to prevent the adoption of ULBs and identify potential individual risk factors. Specifically, emotional and attention dysregulation, as well as impulsivity, have been linked to early onset and chronicity of ULBs such as smoking, substance misuse, unhealthy dietary habits, internet addiction and low quality of sleep.^7,8^ The mentioned individual factors characterise attention-deficit hyperactivity disorder (ADHD),^9^ which affects 12.5% of adolescents between 12 and 18 years old,^10^ whereas a more heterogeneous prevalence of subclinical ADHD, ranging from 0.8 to 23.1% among children and adolescents, has been found.^11^ Although the relevance of subclinical symptoms has been frequently disregarded,^12^ moderate ADHD symptoms in adolescence have been associated with risk of obesity,^13^ smoking^14^ and worse health-related quality of life (QoL).^15^ It should thus be necessary to assess subclinical manifestations of ADHD through a clinimetric approach, which enables the evaluation of symptom severity and exploration of psychosocial factors that contribute to individual susceptibility to illness.^16^ Among psychosocial factors, stressful events, allostatic load, health attitudes/behaviour and psychological well-being have been found to be strictly associated with ULBs in adolescents^17^ and adults.^18,19^ Consequently, it appears of utmost importance to detect young adolescents who present with subclinical ADHD symptoms. This would allow for both the interception of prodromal symptoms of a clinical manifestation of ADHD in adulthood, and for intervention before the association between ADHD and ULBs becomes chronic over time, leading to negative physical and mental health outcomes.^4,6^

## Aim of the study

Based on these premises, this study endeavoured to explore prevalence of ULBs, severity of ADHD symptoms and psychosocial factors related to individual vulnerability (i.e. allostatic overload, abnormal illness behaviour, QoL, psychological well-being) among young adolescents, through a clinimetric approach. Additionally, it aimed at assessing the relationship between various degrees of ADHD symptom severity and ULBs, as well as the aforementioned psychosocial factors.

## Method

### Sample

Four-hundred and forty 14-year-old students (54.5% female) attending the first year of upper secondary schools were enrolled. Inclusion criteria were: (a) students attending the selected classes, (b) informed consent signed by both parents or legal guardian(s) and (c) students’ informed assent for participation in the study.

### Design and procedures

**Fig. 1:**
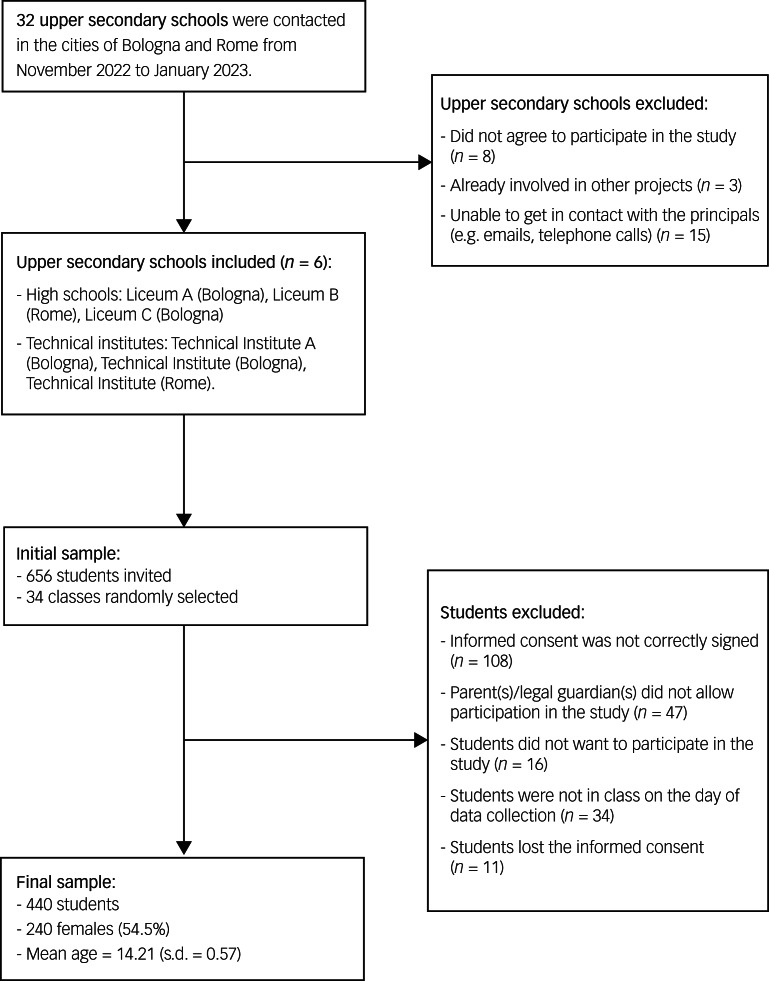
Flow chart of study phases.

Within the scope of the present multicentre cross-sectional study, 32 upper secondary schools were contacted in Bologna and Rome (Italy), from November 2022 to January 2023. Six schools agreed to participate in the study and 34 first-year classes (out of 71) were chosen by means of a random number generator and included in the study, with a student response rate of 67.1% (see Fig. 1 for enrolment flow chart).

During the enrolment period (January 2023 to April 2023), each student was given an informed consent form, which had to be read and signed by both parents or legal guardian(s). Students without signed consent forms were given alternative supervised assignments. All participants’ involvement in the study was anonymous and voluntary, without any payment or compensation (e.g. school credits). The questionnaires were self-administered online through the web version of Microsoft 365 Forms or in paper form in case of missing internet connection/smartphone. The completion of the procedure lasted about 1 h.

The authors assert that all procedures contributing to this work comply with the ethical standards of the relevant national and institutional committees on human experimentation and with the Helsinki Declaration of 1975, as revised in 2013. All procedures involving human participants were approved by the Bioethics Committee of the University of Bologna (protocol number 0369147).

### Assessment

Questions on sociodemographic data (i.e. gender, age, height and weight to calculate body mass index, nationality, members of the household, parents’ level of education/occupation, self-perceived socioeconomic status), *ad hoc* items and validated self-report questionnaires on ULBs, ADHD symptoms and psychosocial factors were administered.

#### Lifestyle

##### Physical activity/sport

*Ad hoc* items on the frequency of 60 min daily physical activity and sport were included.

##### Diet

*Ad hoc* items on adolescents (i.e. regularly consumption of fruits/vegetables and coffee/coke/tea/energy beverages) and their family's (i.e. regularly consumption of fruits/vegetables) dietary habits were included. The Mindful Eating Questionnaire (MEQ),^20^ abbreviated version,^21^ was used to assess consciousness of bodily and emotional sensations experienced when eating. The MEQ includes 20 items rated on a four-point Likert scale encompassing two factors, awareness (on how food affects own internal states; MEQ-AW) and recognition (of sense of hunger/satiety; MEQ-RE). Higher scores indicate greater mindful eating.

##### Alcohol use

The Alcohol Use Disorder Identification Test – Consumption *(*AUDIT-C)^22^ was used to assess problematic alcohol use. The AUDIT-C derives from the original AUDIT^23^ and includes three items rated on a five-point scale of increasing severity. The item on binge drinking was adapted to adolescents, reducing number of drinks from 6 to 5, according to the European School Survey Project on Alcohol and Other Drugs.^24^ Higher total score indicates more severe alcohol use, with a score of ≥3 identifying at-risk drinking.

##### Cigarette use

The two-item Heaviness of Smoking Index (HIS)^25^ was used to assess problematic smoking behaviour. Higher scores indicate more severe smoking habit, with a score of ≥6 identifying cigarette addiction.

##### Cannabis use

The Cannabis Abuse Screening Test (CAST)^26^ was used to assess problematic cannabis use in the whole life, past 12 months and/or 30 days. If respondents report having used cannabis, then six additional items, rated on a five-point Likert scale, evaluate problems related to cannabis use. Higher score indicates more severe cannabis use, with a score of ≥7 suggesting addiction.

##### Other drugs use

Two additional items assessing past and present other drug use were included.

##### Sleep

One item evaluating hours of sleep each night, and another on time spent using technological devices instead of sleeping, were included. Quality of sleep was assessed with an item derived from Pittsburgh Sleep Quality Index,^27^ rated on a four-point Likert scale.

##### Use of technological devices

One item assessing daily time spent using technological devices was included, as well as the Internet Addiction Test (IAT),^28^ to evaluate problematic Internet use. The IAT includes 20 items rated on a five-point Likert scale. The Italian validation study^29^ reported satisfactory psychometric properties and identified six factors: (a) compromised social QoL (i.e. social life impairment resulting from internet use), (b) compromised individual quality of life (i.e. impairment of individuals’ activities resulting from internet use), (c) compensatory usage of the internet (i.e. anticipatory need of internet use), (d) compromised academic/work careers (i.e. academic/work careers impairment resulting from internet use), (e) compromised time control (i.e. incapacity to stop internet use) and (f) excitatory usage of the internet (i.e. excitement associated with going online). Higher scores indicate more severe internet use.

#### Psychological characteristics

##### ADHD symptoms

The Adult ADHD Self-Report Scale (ASRS)^30^ was used to assess ADHD-related symptoms in terms of frequency and severity. The ASRS includes 18 items rated on a five-point Likert scale that allows for a global ADHD score and two scores of ADHD core symptoms: inattention and hyperactivity/impulsivity. Higher scores indicate greater severity of ADHD symptoms. ASRS has been used in adolescents^31^ and demonstrated good psychometric properties (i.e. internal consistency, sensitivity, specificity).^32^

##### Psychosocial factors

The Psychosocial Index – Young (PSI-Y),^33^ derived from the original version of Psychosocial Index,^34^ was used to assess the psychosocial factors of interest. It includes 21 ‘yes/no’ items, 18 items on a four-point Likert scale and a final item assessing QoL on a five-point Likert scale. The operationalisation of allostatic overload, based on specific clinimetric criteria,^16^ requires the presence of an identifiable stressor judged as exceeding or taxing an individual's coping skills (criterion A); and for the stressor to be associated with psychiatric/psychosomatic symptoms, impaired functioning and/or compromised well-being (criterion B). For the current study, allostatic overload was used together with abnormal illness behaviour, which refers to worries about one's own physical health, and a single item on QoL. The PSI-Y showed intraclass correlation coefficients ranging from 0.94 to 0.80.^34^

##### Psychological well-being

The short version of the Psychological Well-Being Scales (PWBs)^35,36^ was used to assess psychological well-being, according to Ryff's multidimensional model encompassing: self-acceptance, positive relationships, purpose in life, environmental mastery, personal growth and autonomy. The questionnaire includes 18 items rated on a four-point Likert scale, with higher scores indicating higher psychological well-being. Internal consistency among the Italian youth population was adequate.^37^

### Statistical analysis

*A priori* power analysis, using G*Power software (version 3.1.9.6 for macOS, Department of Psychology, University of Düsseldorf, Germany; https://www.psychologie.hhu.de/arbeitsgruppen/allgemeine-psychologie-und-arbeitspsychologie/), was conducted to establish the minimum number of participants required. To assess differences between groups, a minimum of 159 participants was required to provide adequate statistical power (1 − *β* = 80%) with *α* = 0.05. Analyses were performed with SPSS (IBM SPSS Statistics for Windows, version 25.0, 2007); *P-*value was set at 0.05.

Descriptive statistics were used to describe the sample. Participants were clustered according to severity of their ADHD symptoms, as follows: ‘no ADHD’ individuals, with an ASRS total score within the second quartile (≤50th percentile); ‘subclinical ADHD’ individuals, with an ASRS total score between the second (≥51st percentile) and third quartile (≤75th percentile); ‘clinical ADHD’ individuals, with an ASRS total score above the third quartile (>75th percentile). To assess differences between the three levels of ADHD symptoms regarding sociodemographic, ULB-related and psychosocial variables, one-way analyses of variance (ANOVAs) and *χ*^2^-tests were used. Regarding ANOVAs, *post hoc* and global (i.e. for multiple measures comparisons between groups) Bonferroni corrections were applied; in particular, global Bonferroni correction was obtained by dividing the standard significance level (*α* = 0.05) by 90 (i.e. 30 dependent variables multiplied for three groups), resulting in an adjusted significance level of 0.00056 (*P* < 0.001). Specifically for *χ*^2^ testing, *post hoc* comparisons were analysed by considering two groups each time (i.e. clinical ADHD versus subclinical ADHD; clinical ADHD versus no ADHD; subclinical ADHD versus no ADHD). Missing data were handled by complete-case analysis; namely, only the cases with complete data were analysed, and individuals with missing data on any of the included variables were dropped from the analyses.

## Results

### Description of the sample

Detailed descriptive statistics are illustrated in Table 1. Of the total sample, 8.6% had a body mass index >25. A total of 10.9% reported physical inactivity and 23.6% did not play any sport. Regarding diet, 19.3% did not consume fruits and vegetables regularly, whereas 75.9% reported drinking energy beverages. Alcohol represented the most used substance (49.3%). Moreover, 12.7% of the sample reported problematic drinking and 14.5% reported binge drinking episodes. Adolescents spent most of their time using a smartphone (mean daily hours: 15.7) and computer (mean daily hours: 8.1).

**Table 1:** Descriptive statistics of the sample (*N* = 440)

Variables	*n*/mean	%/(s.d.)
Gender (female)	240	54.5%
Age	14.21	(0.57)
Nationality (Italian)	399	90.7%
Family socioeconomic status		
Poor	30	6.8%
Middle	332	75.5%
Rich	78	17.7%
Upper secondary school attended		
High school	313	71.1%
Technical institute	127	28.9%
Living with family members	363	82.4%
Body mass index, kg/m^2^	22.82	(31.55)
Missing	7	1.6%
<18.5	127	28.9%
18.5 to <25	268	60.9%
25 to <30	30	6.8%
≥30	8	1.8%
Physical activity		
Everyday	193	43.9%
Every now and then	199	45.2%
Never	48	10.9%
Sport	336	76.4%
Regular fruit/vegetable consumption	355	80.7%
Regular family fruit/vegetable consumption	339	77%
Energy drink consumption	334	75.9%
Mindful Eating Questionnaire		
Awareness	26.48	(5.42)
Recognition	25.46	(4.57)
Daily hours of sleep	6.90	(1.21)
Sleep quality	2.72	(0.77)
Daily hours spent in:		
Watching television	7.67	(82.23)
Listening to the radio	5.00	(67.33)
Using smartphone	15.67	(105.57)
Using tablet	7.45	(82.26)
Using computer	8.08	(82.21)
Playing video games	7.65	(82.24)
Using technological devices instead of sleeping	1.01	(1.32)
Internet Addiction Test	14.02	(3.94)
Compromised social quality of life	14.99	(4.67)
Compromised individual quality of life	10.27	(4.05)
Compensatory usage of internet	5.96	(2.21)
Compromised academic/work career	4.87	(2.15)
Compromised time control	6.01	(1.82)
Excitatory usage of internet	4.10	(1.78)
Cigarette use	68	15.5%
Electronic cigarette use	78	17.7%
Alcohol use	217	49.3%
Binge drinking	82	14.5%
Cannabis use	38	3%
Current drug use	7	1.6%
Past drug use	14	3.2%
Adult ADHD Self-Report Scale	29.82	(10.46)
Inattention	16.03	(6.18)
Hyperactivity/impulsivity	13.78	(5.54)
ADHD symptom severity		
No ADHD	254	57.7%
Subclinical ADHD	97	22%
Clinical ADHD	89	20.2%
Psychosocial Index – Young		
Allostatic overload	272	61.8%
Abnormal illness behaviour	1.65	(2.13)
Quality of life	3.02	(1.33)
Psychological Well-Being scales		
Self-acceptance	7.93	(2.35)
Autonomy	8.42	(2.13)
Environmental mastery	7.92	(2.19)
Personal growth	8.93	(2.12)
Positive relationship	8.74	(2.31)
Purpose in Life	7.99	(2.21)

ADHD, attention-deficit hyperactivity disorder.

Ninety-seven students (22%) were categorised as subclinical ADHD and 89 (20.2%) as clinical ADHD. More than half of the participants (61.8%) reported allostatic overload.

### ADHD symptom severity and ULBs

The MEQ-RE (*P* < 0.001), quality of sleep (*P* < 0.001), IAT total score (*P* < 0.001) and compromised social QoL (*P* < 0.001) were significantly different between the three groups based on ADHD symptom severity, with clinical ADHD reporting greater impairments. Moreover, *post hoc* analyses indicated that compared with no ADHD, both subclinical and clinical ADHD groups reported significantly higher scores in MEQ-AW and in five IAT dimensions (i.e. compromised individual QoL, compensatory usage of the internet, compromised academic/work careers, compromised time control, excitatory usage of the internet), meaning that students with subclinical ADHD showed similar scores as peers with clinical ADHD symptoms. Further, compared with no ADHD, only the clinical ADHD group reported significantly worse outcomes in hours spent using technological devices instead of sleeping, and the clinical ADHD group also reported significantly worse outcomes than both the subclinical and no ADHD groups in two ULBs (i.e. mean daily hours of sleep; hours spent using a smartphone). See Table 2 for detailed results.

**Table 2:** Differences in continuous variables based on attention-deficit hyperactivity disorder (ADHD) symptoms severity

Variables	No ADHD (*n* = 254)	Subclinical ADHD (*n* = 97)	Clinical ADHD (*n* = 89)	*P-*value
Body mass index, kg/m^2^	20.34	20.34	20.80	0.35
Mindful Eating Questionnaire – Awareness	25.62^a^	27.19^b^	28.10^b^	<0.001
Mindful Eating Questionnaire – Recognition	**26**.**42**	**25**.**12**	**23**.**05**	<0.001
Alcohol Use Disorder Identification Test				
Consumption total score	1.31	1.46	2.00	<0.05
Heaviness of Smoking Index	0.56	0.53	1.13	0.25
Cannabis Abuse Screening Test	3.71	6.25	6.84	0.12
Daily hours of sleep	7.11^a^	6.97^a^	6.22^b^	<0.001
Hours using technological devices instead of sleeping	0.78^a^	1.15^a,b^	1.50^b^	<0.001
Quality of sleep^c^	**241**.**56**	**209**.**29**	**169**.**56**	<0.001
Daily hours daily spent in:				
Watching television	0.88	0.75	0.95	0.41
Listening to the radio	0.35	0.66	0.54	0.57
Using smartphone	3.95^a^	4.39^a^	5.55^b^	<0.001
Using tablet	0.43	1.17	0.69	<0.01
Using computer	1.17	1.30	1.57	0.13
Playing video games	0.91	0.66	0.87	0.38
Internet Addiction Test, total score	**12**.**48**	**15**.**49**	**16**.**81**	<0.001
Compromised social quality of life	**13**.**67**	**15**.**95**	**17**.**67**	<0.001
Compromised individual quality of life	8.49^a^	11.46^b^	12.74^b^	<0.001
Compensatory usage of internet	5.36^a^	6.57^b^	6.97^b^	<0.001
Compromised academic/work career	4.19^a^	5.53^b^	6.07^b^	<0.001
Compromised time control	5.48^a^	6.52^b^	6.92^b^	<0.001
Excitatory usage of internet	3.57^a^	4.62^b^	5.00^b^	<0.001
Psychosocial Index – Young				
Abnormal illness behaviour	**1**.**13**	**1**.**94**	**2**.**78**	<0.001
Quality of life	**2**.**81**	**2**.**52**	**2**.**21**	<0.001
Psychological Well-Being scales				
Environmental mastery	8.65^a^	7.17^b^	6.62^b^	<0.001
Autonomy	8.81^a^	8.12^b^	7.59^b^	<0.001
Personal growth	9.06	8.75	8.71	0.27
Positive relations	9.11^a^	8.36^b^	8.05^b^	<0.001
Purpose in life	8.55^a^	7.40^b^	7.01^b^	<0.001
Self-acceptance	**8**.**48**	**7**.**55**	**6**.**75**	<0.001

Global Bonferroni correction for multiple measures comparisons between groups [*P* = 0.05/(30×3)] was applied. Mean values in bold indicate that all groups are significantly different from each other (*P* < 0.05).

a. Mean values sharing the same superscript are not significantly different from each other.

b. Mean values with different superscripts are significantly different from each other (*P* < 0.05).

c. Mean-rank values compared with Kruskal–Wallis test and Dwass–Steel–Critchlow–Fligner pairwise comparisons.

Chi-squared analyses indicated that no categorical ULB was significantly different between the three ADHD groups. However, *post hoc* comparisons indicated that compared with the no ADHD group, only the clinical ADHD group reported significantly worse outcomes in five ULBs (i.e. sport activity; family fruit and vegetable consumption; tobacco smoke; electronic cigarette use; past drug use), and the clinical ADHD group also reported a significantly higher frequency of binge drinking than both subclinical and no ADHD groups. See Table 3 for detailed results.

**Table 3:** Differences in categorical variables based on attention-deficit hyperactivity disorder (ADHD) symptoms severity

Variables	No ADHD (*n* = 254)	Subclinical ADHD (*n* = 97)	Clinical ADHD (*n* = 89)	*χ*^2^
Physical activity				
Sometimes	42.1%	49.5%	49.4%	8.34
Everyday	48.4%	41.2%	33.7%	8.34
Sport	81%^b^	76.3%^a^^,^^b^	64.8%^a^	9.66*
Fruits/vegetable consumption	84.3%	77.3%	74.2%	5.21
Family fruit/vegetable consumption	83.1%^b^	74.2%^a^^,^^b^	62.9%^a^	15.68**
Energy drink consumption	73.2%	76.3%	83.1%	3.55
Binge drinking	14.2%^b^	18.6%^b^	31.5%^a^	12.99*
Cigarette use	11.8%^b^	15.5%^a^^,^^b^	25.8%^a^	9.93*
Electronic cigarette use	13.8%^b^	19.6%^a^^,^^b^	27%^a^	8.15*
Past drug use	1.2%^b^	4.1%^a^^,^^b^	7.9%^a^	9.91*
Current drug use	0.8%	3.1%	2.2%	2.69
Allostatic overload	52%^b^	73.2%^a^	77.5%^a^	25.07**

a. Frequencies sharing the same superscript are not significantly different from each other.

b. Frequencies with different superscripts are significantly different from each other (*P* < 0.05).

*P < 0.01; **P < 0.001.

### ADHD symptom severity and psychosocial factors

Allostatic overload was significantly more frequent (*P* < 0.001) in both subclinical (73.2%) and clinical (77.5%) ADHD groups, compared with the no ADHD group (52%) (Table 3).

Regarding PSI-Y and PWB dimensions, PSI-Y abnormal illness behaviour (*P* < 0.001), QoL (*P* < 0.001) and PWB self-acceptance (*P* < 0.001) scores were significantly different between the three groups, with the clinical ADHD group reporting the worst scores. Moreover, compared with the no ADHD group, both the subclinical and clinical ADHD groups showed significant lower scores in four PWB dimensions (i.e. environmental mastery, autonomy, positive relations, purpose in life) (Table 2).

## Discussion

The present study aimed to examine how common ULBs and ADHD symptoms are among young adolescents, as well as investigate differences between groups based on ADHD symptom severity in relation to ULBs and specific psychosocial factors. Results support literature showing a strict association between clinical ADHD, ULBs and psychosocial impairments. The most relevant finding of the present investigation, however, is that subclinical ADHD symptoms are also associated with several ULBs and similar psychosocial dysfunctions. To the best of our knowledge, the present multi-centre study is the first to evaluate a comprehensive range of ULBs in relation to different ADHD severity, in a sample of students attending first year of upper secondary schools.

Consistent with previous research, current findings indicated that ULBs, such as unhealthy eating behaviours, alcohol use, problematic technology use and sleep problems, are quite common among adolescents.^38,39^ These behaviours may be influenced by social pressure from peers,^1^ which can lead individuals to identify with a crowd and adopt its norms and behaviours, and possibly ULBs, to feel part of the group.

The present investigation found that 20.2% of 14-year-old adolescents reported clinical ADHD symptoms, a higher rate than in previous studies.^40^ However, ADHD prevalence greatly varies depending on the diagnostic criteria used.^10^ Conversely, the prevalence of subclinical ADHD symptoms found in the present study (22%) is in line with a review indicating a prevalence between 0.8 and 23.1%.^11^

As expected, adolescents with clinical ADHD symptoms reported the greatest impairments in the majority of ULBs (i.e. MEQ-RE, IAT total score, IAT compromised social QoL, quality and mean hours of sleep, time spent using smartphones, use of technological devices instead of sleeping) and psychosocial factors (i.e. PSI-Y and PWB dimensions). These results are consistent with research linking ADHD to the adoption of ULBs^41,42^ and worse health-related QoL.^15^ It has been hypothesised that sensation seeking, emotional dysregulation and psychological distress, all aspects characterising ADHD, may be leading causes of ULBs.^43,44^ Regarding mindful eating, although our analyses highlighted that ADHD is associated with higher MEQ-AW scores, this finding does not necessarily reflect healthier behaviours among adolescents with ADHD symptoms. Awareness, according to the MEQ, refers to being aware of how food affects senses and internal states.^21^ Since literature demonstrated that ADHD is strongly associated with emotional lability^45^ and that negative affectivity and emotional dysregulation mediate the relationship between ADHD and disordered eating (e.g. by triggering abnormal food intake),^43^ the present results are not unexpected. Indeed, higher scores on the MEQ-AW could be explained by the fact that adolescents with ADHD symptoms seem to be aware how food affects their own internal states, and possibly helps them to cope with emotional lability. Although further studies aimed at a better understanding of the connection between ADHD-related emotional dysregulation and awareness on eating habits are necessary, the current findings imply that future interventions should focus on preventing/reducing the risk of developing unhealthy eating behaviours (e.g. those leading to obesity) in adolescents. Furthermore, these interventions might be more effective if they take into account the level of awareness on eating habits among adolescents exhibiting ADHD symptoms.

The most relevant finding of the present study is represented by the fact that also students with subclinical ADHD symptoms reported significant impairments in several ULBs and psychosocial factors. Indeed, imbalanced awareness and recognition of eating behaviours (i.e. the MEQ), problematic internet use (i.e. the IAT), low quality of sleep and impaired psychosocial factors (i.e. greater frequency of allostatic overload, abnormal illness behaviour, lower QoL, environmental mastery, autonomy, positive relationships, purpose in life and self-acceptance) are also significantly associated with subclinical ADHD symptoms. This suggests that although ADHD symptoms may not be severe enough to meet criteria for clinical ADHD, they have a meaningful impact on numerous aspects of daily life anyway. Research has shown that having ADHD during adolescence can lead to the development of ULBs^41^ and more severe mental health issues in adulthood.^46^ Although there is still uncertainty about whether ADHD can be diagnosed in adulthood without a prior diagnosis during childhood or adolescence,^47^ it has been proposed that subclinical symptoms of ADHD may be present during childhood and adolescence, becoming clinically significant only in adulthood.^48^ One possible explanation for underdiagnosed ADHD could be that during adolescence, subclinical ADHD symptoms may be misdiagnosed or overlooked because of the adoption of ULBs used as coping mechanisms to manage symptoms of emotional dysregulation.^49^ Therefore, correctly identifying and addressing subclinical ADHD symptoms in adolescence can help to prevent the adoption of ULBs and reduce the likelihood of a late diagnosis of clinical ADHD.

Finally, regarding psychosocial factors, it was found that allostatic overload is highly prevalent among young adolescents, especially among those with either clinical or subclinical ADHD symptoms. This finding is in line with previous literature highlighting how stress is common among adolescents with ADHD, negatively affects well-being and is usually linked to feelings of helplessness and overwhelm (and thus incapacity to manage stress), ill health and anxiety.^50^ It has been hypothesised that emotional dysregulation (e.g. poor temper control, emotional lability, emotional over-reactivity, hyperactivity/restlessness), which often affects people with ADHD, could account for a diminished ability to manage typical life stressors,^50,51^ negatively influencing social functioning.^52^ As to abnormal illness behaviours and QoL, individuals with both clinical and subclinical ADHD symptoms seem to be characterised by more severe hypochondriac beliefs, bodily preoccupations and lower QoL, in line with existing literature.^15,53^ The present findings support the idea that ADHD symptoms is linked to poorer outcomes in terms of psychological well-being, consistent with previous research.^54^ Both clinical and subclinical ADHD groups show similar levels of impaired psychosocial functioning. Although there are no studies demonstrating these specific associations for subclinical ADHD, it is possible that the presence of psychopathological processes like rumination and sensation seeking, typically associated with ADHD,^9,50^ may already be present in subclinical symptoms and could affect psychosocial functioning.

There are several limitations to the present study. First, the reliance on self-report measures could introduce biases resulting from social desirability. Second, given that only self-report measures were employed, ADHD cannot be clinically diagnosed in the current sample. Future studies should investigate ULBs in cohorts diagnosed with ADHD according to the DSM. Finally, there is an ongoing debate regarding how to determine subclinical ADHD^11^ and the clinical utility of the proposed cut-offs,^55^ as specific criteria have not been established yet, which may have influenced the prevalence of subclinical symptoms in the current study. Future research should strive to develop a standardised and shared approach for identifying subclinical ADHD symptoms.

The current study has also relevant strengths. First, the comprehensive assessment allowed for the identification of different levels of ADHD severity and their association with ULBs and specific psychosocial factors (i.e. allostatic overload, abnormal illness behaviour, impaired QoL and psychological well-being) that have been proven risk factors in other settings,^19^ but have not yet been considered in this context, providing valuable new insights. In addition, whereas previous studies usually included wider age ranges (possibly affecting effect sizes of the associations between variables), this investigation focused specifically on 14-year-old adolescents. This allowed us to identify unique patterns of risky behaviours in this age group, which differ from those seen in younger or older individuals.

Our findings may have relevant clinical implications. First, the clinimetric assessment allowed for the identification of a variety of ULBs, different levels of ADHD severity and psychosocial factors. Compared with traditional taxonomies, this approach provides more precise indications of which aspects of everyday life are more impaired among adolescents with ADHD-related symptoms. Second, it is essential to focus and correctly identify subthreshold manifestations of ADHD, even in non-clinical settings where they may be hidden by specific ULBs. Prompt evaluations may reduce the likelihood of worse mental and physical outcomes in adulthood (e.g. worsening of clinical ADHD symptoms, need of a pharmacological treatment potentially associated with detrimental long-term effects,^56^ and chronicity of ULBs), and may support clinicians to provide tailored interventions addressing needs of vulnerable adolescents. This comprehensive understanding may improve diagnosis accuracy, treatment efficacy and ultimately enhance the overall well-being of this population group.

## Data Availability

The data that support the findings of this study are available from the corresponding author, S.G., upon reasonable request.
